# Perishing for Lack of Knowledge

**Published:** 1871-02

**Authors:** 


Perishing for Lack of Knowledge.—In Great Britain alone more than one hundred thousand people perish annually, and at least five times as many sicken grivously, out of pure ignorance of the laws of health, which are never imparted to them at school. They have no chance of learning them afterward, as they possess no secondary schools. The mere tools of education are put into the hands of children during their school time, without any effort being made to teach them how to use the tools for any profitable purpose whatever.

In Great Britain statistics are more carefully taken regarding these subjects than in America, but it is probable that here there is quite as much loss of life and time, from ignorance of the laws of health, as there. If we are to judge of the general diffusion of sanitary knowledge in England by the sale of books on health, they certainly read more on the subject than we do.—*Herald of Health.*

